# Modulation of Litter Decomposition by the Soil Microbial Food Web Under Influence of Land Use Change

**DOI:** 10.3389/fmicb.2018.02860

**Published:** 2018-11-26

**Authors:** Amber Heijboer, Peter C. de Ruiter, Paul L. E. Bodelier, George A. Kowalchuk

**Affiliations:** ^1^Biometris, Wageningen University & Research, Wageningen, Netherlands; ^2^Institute of Environmental Biology, Utrecht University, Utrecht, Netherlands; ^3^Institute for Biodiversity and Ecosystem Dynamics, University of Amsterdam, Amsterdam, Netherlands; ^4^Department of Microbial Ecology, Netherlands Institute of Ecology (NIOO-KNAW), Wageningen, Netherlands

**Keywords:** soil microbial community, decomposition, PLFA-SIP, carbon cycle, agricultural abandonment, soil food web

## Abstract

Soil microbial communities modulate soil organic matter (SOM) dynamics by catalyzing litter decomposition. However, our understanding of how litter-derived carbon (C) flows through the microbial portion of the soil food web is far from comprehensive. This information is necessary to facilitate reliable predictions of soil C cycling and sequestration in response to a changing environment such as land use change in the form of agricultural abandonment. To examine the flow of litter-derived C through the soil microbial food web and it’s response to land use change, we carried out an incubation experiment with soils from six fields; three recently abandoned and three long term abandoned fields. In these soils, the fate of ^13^C-labeled plant litter was followed by analyzing phospholipid fatty acids (PLFA) over a period of 56 days. The litter-amended soils were sampled over time to measure ^13^CO_2_ and mineral N dynamics. Microbial ^13^C-incorporation patterns revealed a clear succession of microbial groups during litter decomposition. Fungi were first to incorporate ^13^C-label, followed by G^−^ bacteria, G^+^ bacteria, actinomycetes and micro-fauna. The order in which various microbial groups responded to litter decomposition was similar across all the fields examined, with no clear distinction between recent and long-term abandoned soils. Although the microbial biomass was initially higher in long-term abandoned soils, the net amount of ^13^C-labeled litter that was incorporated by the soil microbial community was ultimately comparable between recent and long-term abandoned fields. In relative terms, this means there was a higher efficiency of litter-derived ^13^C-incorporation in recent abandoned soil microbial communities compared to long-term abandoned soils, most likely due to a net shift from SOM-derived C toward root-derived C input in the soil microbial food web following land-abandonment.

## Introduction

Soils receive their carbon (C) via two major pathways: the “green” pathway via readily available plant root exudates and the “brown” pathway via slowly decomposing dead plant material in the form of litter (Moore et al., 2005). Since most of the soil C input is originating from the plant detritus production, litter fall has a large influence on the soil food web in terms of mineralization processes, nutrient cycling and the formation and sequestration of organic matter (Gessner et al., 2010; van der Wal and de Boer, 2017). Soil microbial organisms form the first detritus consuming trophic level are responsible for the majority of soil mineralization (de Ruiter et al., 1994). Soil microbes have therefore been recognized to function as the “eye of the needle” through which eventually all organic matter passes (Jenkinson, 1977), thereby determining to a large extent the C partitioning between mineralization (CO_2_ evolution) and sequestration. The activity, biomass and composition of soil microbial communities are largely influenced by changes in the composition of organic pools (Heijboer et al., 2016), which are known to be significantly affected by land use change (Degryze et al., 2004; Leifeld and Kögel-Knabner, 2005).

Despite the recognition of microbes as important contributors to soil food web functioning, it is still largely unknown what the timing and relative contribution of distinct soil microbial functional groups to different phases of litter decomposition is. So how can we increase the level of detail in our knowledge about the microbial part of the soil food web to better understand and predict the effects of land use change on carbon cycling? The introduction of stable isotope probing (SIP) into studies of microbial decomposition offers the possibility to follow the fate of labeled litter through the soil food web (Morriën, 2016). SIP was for instance used to show that G^−^ bacteria typically are associated with the consumption of easily degradable substrates (Treonis et al., 2004; Creamer et al., 2016), while G^+^ bacteria appear to be more important in the decomposition of SOM (Kramer and Gleixner, 2006, 2008). Interestingly, SIP-based experiments have also suggested saprotrophic fungi as major players in the rapid processing of easily available root C (Pausch et al., 2016). Even though there is information about the functional roles of different microbial groups in litter decomposition, a full analyses of the relative timing and activity of different microbial groups, and C flows during the decomposition of a complex realistic substrate like litter is generally missing (Moore-Kucera and Dick, 2008). This type of information on decomposition processes can be of great value in understanding the effects of land use change on global C cycling, and will help to improve existing soil food web models with the ultimate aim of predicting soil function and C turnover in changing environments.

A common form of land use change that has been extensively studied is agricultural land abandonment (e.g., van der Wal et al., 2006; Holtkamp et al., 2008, 2011; Morriën et al., 2017). Therefore we examined soil microbial litter decomposition in six ex-arable soils. The studied ex-arable fields, three long-term and three recently abandoned soils, are part of a well-studied chronosequence and known to differ in mineralization rates and microbial community structure (van der Wal et al., 2006; Holtkamp et al., 2011; Morriën et al., 2017). For the selected fields, Morriën et al. (2017) have shown that a tightening of the belowground soil food webs following land abandonment is associated with increased nutrient cycling and carbon uptake. This effect is argued to be caused by a shift in activity and composition of the fungal community that is active in the processing of “green” (rhizosphere-derived) C. The majority of C input in these ex-arable fields is, however, via the “brown” pathway (detritus-derived) (Holtkamp et al., 2008). The main research aim of this study was therefore to examine how soil microbial communities differ between recent and long-term abandoned soils in response to litter addition and to examine which functional groups of the soil microbial food web are actively involved in the decomposition of organic matter at different phases of the decomposition process. We hypothesized that litter decomposition activity of the soil microbial community would increase after land abandonment, because of a relative increase in the activity and efficiency of soil fungi, with fungi being better equipped to decompose complex litter. We further hypothesized that during litter decomposition the sequence of active microbial groups, e.g., the topological structure of the soil microbial food web, would remain similar across different land abandonment stages.

## Materials and Methods

### Site Description and Soil Sampling

Soil samples were collected from six ex-arable fields, all located on the same glacial sandy soil deposits in the center of Netherlands (Veluwe). All fields had a similar previous crop-rotation history, including barley and potato, and are part of a well-described chronosequence of ex-arable fields (Kardol et al., 2005; van der Wal et al., 2006; Holtkamp et al., 2008; Morriën et al., 2017). After agricultural abandonment, all fields were grazed and annually mowed which allowed the establishment of semi-natural grasslands. Three of the sampled fields are referred to as recently abandoned fields, since they have been abandoned from agricultural practices for 5–9 years. The other field sites have been abandoned for 29–32 years and are therefore referred to as long-term abandoned fields. Details of these study sites are given in Table 1. Soil sampling took place in October 2014 by collecting eight soil samples (10 cm deep, 6 cm diameter) per ex-arable field. Soil samples were taken at least 20 m from the edge of a field, and at least 10 m apart. Aboveground vegetation was removed prior to transportation. Soil samples were transported and stored at 4°C for a week prior to further treatment.

**Table 1:** Overview of ex-arable field sites where soil samples were taken, including information on the exact location, land abandonment stage and time of abandonment of agricultural practices.

Site	Location	Land abandonment	Abandoned since
TW – Telefoonweg	N 52°00′9 E 5°45′8	Recent	2009
RK – Reijerskamp	N 52°1′0 E 5°46′21	Recent	2005
OR – Oud Reemst	N 52°2′27 E 5°48′34	Recent	2005
DK – Dennenkamp	N 52°1′43 E 5°48′2	Long-term	1982
MV – Mosselse Veld	N 52°4′23 E 5°44′13	Long-term	1985
BB – Boersbos	N 52°3′44 E 5°59′57	Long-term	1982

### Production of ^13^C-Labeled Litter

In the summer of 2012, ten soil cores (12 cm diameter, 20 cm deep) were collected per field site, including standing vegetation. Nine of those cores were transported to the laboratory and labeled with 99.99 atom% ^13^CO_2_ (Cambridge Isotope Laboratories, Andover, MA, United States) for 13 h; for details see (Morriën et al., 2017). The remaining core served as a non-labeled control core. Aboveground biomass was harvested, freeze dried and ground (Retsch ZM100, Haan, Germany). All aboveground biomass coming from labeled cores was combined into a composite batch and subsequently the δ^13^C value of both labeled and unlabeled ground plant material was measured on an elemental analyzer (Flash2000, Thermo) attached to an isotope ratio mass spectrometer (IRMS, Thermo) and revealed an average δ^13^C of 904.0 and −25.7 for, respectively, ^13^C-labeled and unlabeled control plant material. The labeled plant material was used as a ^13^C-labeled substrate in the incubation experiment (see below) and is further referred to as ^13^C-labeled litter.

### Experimental Design:^13^C Incubation Experiment

Soil samples were sieved (2 mm) to remove roots and stones and subsequently pooled into one composite soil sample per field site. For each of the six field sites, a total of 30 glass bottles of 333 ml were filled with an equivalent of 50 g of dry weight soil. Three out of every five bottles were supplemented with 0.5 g of ^13^C-labeled litter, one out of every five bottles received 0.5 g of unlabeled litter (positive control, C^+^) and the remaining bottles received no litter (negative control, C^−^). C inputs corresponded to 10–15% of the total organic matter C in the fields used in our study (Holtkamp et al., 2008). MilliQ water was added to the composite soil/litter samples until a WHC of 60% was reached. The bottles were closed with a cotton ball to avoid contamination, while permitting gas exchange and incubated in the dark at 17.5°C. One-sixth of all bottles was destructively sampled at each of the following time points: 1, 3, 7, 14, 28, and 56 days. Per field site, this corresponded to three labeled samples (technical replicates), plus a positive and negative control sample. A subsample of each soil (equivalent to 5 g dry weight of soil) was stored at 4°C prior to the determination of extractable nutrients. Remaining soil was stored in the freezer at −80°C prior until further analyses.

### ^13^CO_2_ Respiration Rates

During the entire experimental period of 56 days, ^13^CO_2_ flux measurements were taken. The 30 bottles that were harvested on day 56 were subjected to gas sampling throughout the entire experiment. On day 1, 3, 7, 14, 28, and 55 (because of destructive sampling on day 56), the cotton balls were removed from the bottles and subsequently closed with a lid with a rubber septum. Directly after closure, an air sample of 15 ml was taken from the headspace (*t* = 0) using a 25 ml analytical syringe (SGE Analytical Science, Australia), and stored in a 12 ml Labco Exetainer^®^ 12 ml vial. The headspace air pressure-drop was compensated by injecting 15 ml of N_2_ gas. A second gas sample was taken after ±4.5 h (*t* = 1). Gas samples were analyzed on a Thermo Trace Ultra GC interfaced with a methanizer-FID or a Thermo Scientific Delta V IRMS to determine, respectively, CO_2_ and δ^13^CO_2_ values (Rt-Q-BOND column; 30 m, 0.32 mm id, 10 μm film thickness). The CO_2_ concentration and δ^13^CO_2_ values at *t* = 0 and *t* = 1 were used to calculate the CO_2_ and ^13^CO_2_ respiration rates on each sampling date.

### Extraction and Analyses of PLFA

Phospholipid fatty acids (PLFAs) were extracted from 4 g of each soil sample following the procedure of Frostegård et al. (1993) and Hedlund (2002), based on the method of Bligh and Dyer (1959) and White et al. (1979). PLFA extractions were analyzed on a gas chromatograph (GC-FID, 7890A, Agilent technologies, Delaware, United States) to determine the abundance of PLFA biomarkers. The δ^13^C value for each PLFA biomarker was determined by analyzing PLFA extractions on a Thermo Trace Ultra GC, interfaced with a Thermo Scientific Delta V IRMS. For both GC analyses an Agilent HP-5MS UI column (60 m, 0.25 mm id, 0.25 μm film thickness) was used. PLFA biomarkers were used to characterize different microbial groups: fungi (18:2ω6), G^−^ bacteria (16:1ω7, cy17:0, 18:1ω7), G^+^ bacteria (a15:0, a17:0, i15:0, i16:0, i17:0), actinomycetes (10Me16:0, 10Me17:0, 10Me18:0) and microfauna (20:4, Wilkinson et al., 2002). A total number of 24 PLFA biomarkers were used to study soil microbial community composition and to calculate total microbial biomass. The actual δ^13^C value of each PLFA biomarker was calculated as described by Boschker (2004):

(1) $$\delta^{13}C_{\mathrm{PLFA}}=((n+1)*\delta^{13}C_{\mathrm{FAME}}-1*\delta^{13}C_{\mathrm{methanol}})/n$$

where n is the number of C atoms in the PLFA biomarker. The δ^13^C_*PLFA*_ of labeled and unlabeled control samples was used to calculate the actual excess amount of ^13^C in each PLFA biomarker (Boschker, 2004).

### Extractable Nutrient Analysis

Within 24 h of sampling, soil samples were extracted with 100 ml 0.2 M KCl. Samples were shaken for 1 h at 120 rpm on a rotary shaker (Laboshake, Gerhardt, Germany). Samples were centrifuged for 4 min at 4000 rpm (Megafuge 1.0R, Heraeus Instruments, Germany) and filtrated with glass fiber filters (Whatman GF/C, Whatman, Germany). All samples were analyzed spectrophotometrically for NO_3_-N and NH_4_^+^-N on a segmented flow analyzer (Skalar SanPlus, Skalar Analytical, Netherlands). Total available N content was calculated by summing NO_3_-N and NH_4_^+^-N.

### Calculations of Litter-Derived C

To calculate the relative amounts of PLFA and CO_2_ that were litter-derived, the following formula was used:

(2) $$\%C_{\mathrm{litter}}=\frac{\delta^{13}C_{\mathrm{sample}}-\delta^{13}C_{\mathrm{reference}}}{\delta^{13}C_{\mathrm{litter}}-\delta^{13}C_{\mathrm{soil}}}*100$$

where δ^13^C_sample_ refers to the tested sample, δ^13^C_reference_ to the corresponding control sample without litter addition and δ^13^C_litter_ and δ^13^C_soil_, respectively, to the added litter and soil at the start of the experiment.

### Data Analysis and Statistics

Results were analyzed using R version 3.1.0 (R Core Team, 2014). Mineral N, CO_2_, and PLFA abundances, excess^13^C and %C_litter_ differences between land abandonment stages and over time were tested using linear-mixed effect modeling (*nlme* package, Pinheiro et al., 2017), combined with the *car* package (Fox and Weisberg, 2011) for type II sum of squares with “Field” as a fixed factor. Shapiro’s test was used to check normality of residuals, Levene’s test (*car* package) to test for heterogeneity of variances, and data was log or power transformed where necessary.

Abundances, ^13^C-excess and %litter-derived of individual PLFA biomarkers were used as input values for the principal component analysis (PCA) to check for differences in (functional) microbial community structure between different fields and the two land abandonment stages, whereby indicative microbial groups were plotted in a PCA diagram.

To visualize the sequential order of microbial groups throughout the first stages of litter decomposition, the excess amount of ^13^C in different microbial groups was normalized over time using the following formula:

(3) $${\mathrm{Normalized Excess}}^{13}C=\frac{{\mathrm{Excess}}^{13}\mathrm{C-mean}({\mathrm{Excess}}^{13}C)}{\mathrm{sd}({\mathrm{Excess}}^{13}C)}$$

where Excess^13^C is the excess amount of ^13^C in the PLFA of a specific microbial group at a specific time point, with mean (Excess^13^C) and sd (Excess^13^C), respectively, the average and standard deviation of the excess amount of ^13^C for that same microbial group over the complete incubation time. Linear-mixed-effect modeling was used to test whether normalized Excess^13^C patterns differed between microbial groups over time, with “Field” as a fixed factor. Absolute value transformation was applied to meet homogeneity and normality requirements.

## Results

### Soil Microbial Community Development Following Land Abandonment

**FIGURE 1:**
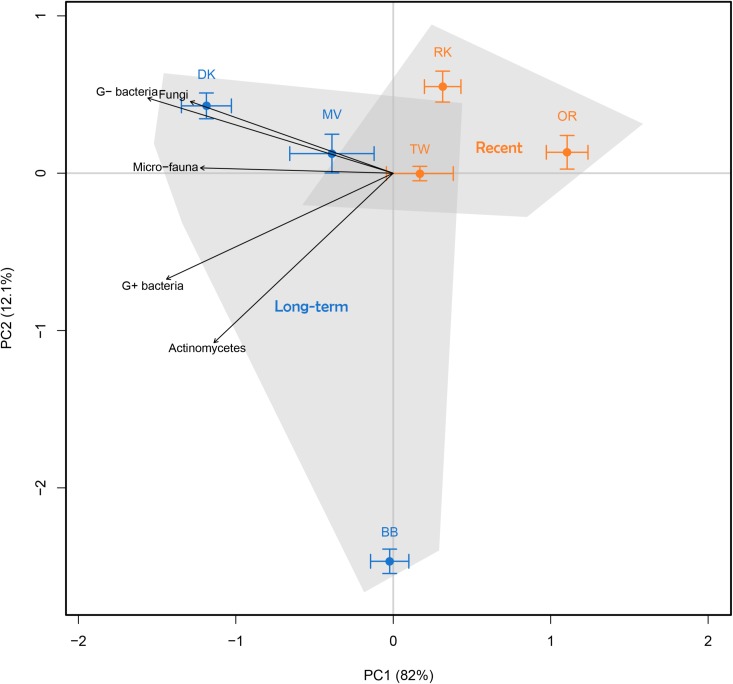
Principal component analysis (PCA) of the relative abundance of soil microbial PLFA biomarkers to characterize soil microbial community composition. All six fields are indicated by mean ± SE, where orange represents recently abandoned fields and blue represents long-term abandoned fields. Arrows show indicative microbial groups (*r*^2^ with *p* < 0.05).

Comparison of all control samples (no litter addition) based on abundance of phospholipid fatty acid (PLFA) biomarkers revealed an effect of land abandonment stage on the composition of the soil microbial community (Figure 1). Long-term abandoned fields exhibited a large degree of variation, although much of this is along the second PCA axis, while the first axis explains the vast majority of the total variation (82%). The microbial communities of the long-term abandoned fields, Dennenkamp (DK) and Mosselse Veld (MV), are strongly characterized by a relatively high abundance of G^−^ bacteria and fungi, while the abundance of micro-fauna, G^+^ bacteria and actinomycetes was more pronounced in soil microbial communities of all long-term abandoned fields. When testing the total abundance of microbial PLFA biomarkers, we observed that there is a significantly higher microbial biomass in long-term abandoned soils compared to recently abandoned soils (χ^2^ = 8.5307, *p* = 0.003). For specific microbial groups, there is a trend of higher abundances in long-term abandoned soils, compared to recently abandoned soils, however, these differences are only significant for G^+^-specific PLFA biomarkers (χ^2^ = 8.5639, *p* = 0.003) and PLFA biomarkers specific to actinomycetes (χ^2^ = 5.6971, *p* = 0.017). The abundance of PLFA biomarkers specific to fungi, G^−^ bacteria and micro-fauna, as well as the fungal/bacterial and G^−^/G^+^ bacterial ratio, were not significantly different between the two land abandonment stages (see Table 2 for a complete overview). When looking at the relative abundances for individual biomarkers, more than half of all biomarkers showed a (marginally) significant higher abundance in long-term abandoned soils compared to recently abandoned soils (Supplementary Table S1).

**Table 2:** Soil microbial community abundances and ratios based on PLFA biomarkers for each land abandonment stage (recent and long-term).

	Land abandonment stage				
	*Recent*	*Long-term*	*χ^2^*	*p*	
Total PLFA (nmol C g soil^−1^)	872.33 ± 35.11	1177.36 ± 37.50	8.4286	0.004	^∗∗^
Fungal PLFA (nmol C g soil^−1^)	18.29 ± 1.34	22.08 ± 1.57	0.8014	0.371	
G^−^ bacterial PLFA (nmol C g soil^−1^)	182.4 ± 10.01	254.94 ± 16.43	1.7844	0.182	
G^+^ bacterial PLFA (nmol C g soil^−1^)	190.23 ± 6.75	261.58 ± 9.09	8.5639	0.003	^∗∗^
Actinomycetes PLFA (nmol C g soil^−1^)	70.71 ± 3.23	110.16 ± 5.99	5.6971	0.017	^∗^
Micro-faunal PLFA (nmol C g soil^−1^)	9.37 ± 0.66	13.03 ± 0.60	2.801	0.094	.
Fungal/bacterial^∗^ PLFA ratio	0.041 ± 0.002	0.035 ± 0.002	2.209	0.137	
G^+^/G^−^ PLFA ratio	1.07 ± 0.03	1.10 ± 0.09	0.098	0.754	

*The first row shows mean ± SE of the total abundance of PLFA biomarker in the soil, subsequent rows show the mean ± SE abundance of PLFA specific biomarkers for different microbial groups. χ^2^ and p-values are the result of linear-mixed modeling (^∗∗∗^p < 0.001, ^∗∗^p < 0.01, ^∗^p < 0.05, ^.^p < 0.1). ^∗^Bacterial PLFA is the sum of G^−^, G^+^, and Actinomycete PLFA.*

### C and N Mineralization Patterns Following Litter Addition

One day after the addition of ^13^C-labeled litter there was more than a tenfold increase in the total amount of respired CO_2_, which gradually decreased back to the control level over the course of the experiment (Table 3). Overall, long-term abandoned soils showed no significant higher CO_2_ respiration compared to recently abandoned soils (χ^2^ = 3.2936, *p* = 0.070). The absolute and relative amounts of litter-derived CO_2_ were not significantly different between the different land abandonment stages, although there were significant interaction effects between land abandonment stage and time. The total amount of mineral N in recently abandoned soils decreased compared to the control treatment until day 7, while the mineral N content for long-term abandoned soils showed an initial increase followed by a similar trend as observed for recently abandoned soils (Table 3). After day 7, the amount of mineral N increased until the end of the incubation experiment (Table 3). No significant differences were found in mineral N dynamics between recent and long-term abandoned soils.

**Table 3:** Carbon and nitrogen mineralization parameters for recent and long-term abandoned ex-arable soils during incubation at each respective sampling moment.

				Time (days)									
*Abiotic measurement*	*Unit*	*Land abandonment stage*	*Control*	*1*	*3*	*7*	*14*	*28*	*56*		*χ^2^*	*p*	
Carbon mineralization	μg C g soil^−1^ day^−1^	Recent	7.177	89.095	52.6	38.347	25.563	16.333	8.762	Stage:	3.2936	0.070	.
		Long-term	8.647	96.569	59.423	39.151	28.939	19.616	6.68	Stage^∗^Time:	7.1933	0.207	
Litter-derived ^13^CO_2_	μg ^13^C g soil^−1^ day^−1^	Recent		45.851	18.15	9.191	2.89	1.369	0.404	Stage:	1.3163	0.251	
		Long-term		50.572	20.407	8.87	4.161	1.804	0.414	Stage^∗^Time:	20.796	<0.001	^∗∗∗^
Litter-derived CO_2_	%	Recent		51.093	34.457	23.805	11.213	8.286	4.583	Stage:	0.2719	0.602	
		Long-term		52.729	34.282	22.355	14.354	9.082	4.245	Stage^∗^Time:	31.578	<0.001	^∗∗∗^
Mineral nitrogen	nmol g soil^−1^	Recent	316.73	243.50	118.70	105.36	240.24	506.94	833.49	Stage:	1.5005	0.221	
		Long-term	164.43	281.90	110.90	118.70	319.08	714.07	898.55	Stage^∗^Time:	0.1335	0.715	

*Control measurements refer to samples without litter addition and were excluded from linear mixed modeling from which χ^2^ and p-values are shown in the latter two columns (^∗∗∗^p < 0.001, ^∗∗^p < 0.01, ^∗^p < 0.05, ^.^p < 0.1).*

### Soil Microbial Community Response to Litter Addition

**FIGURE 2:**
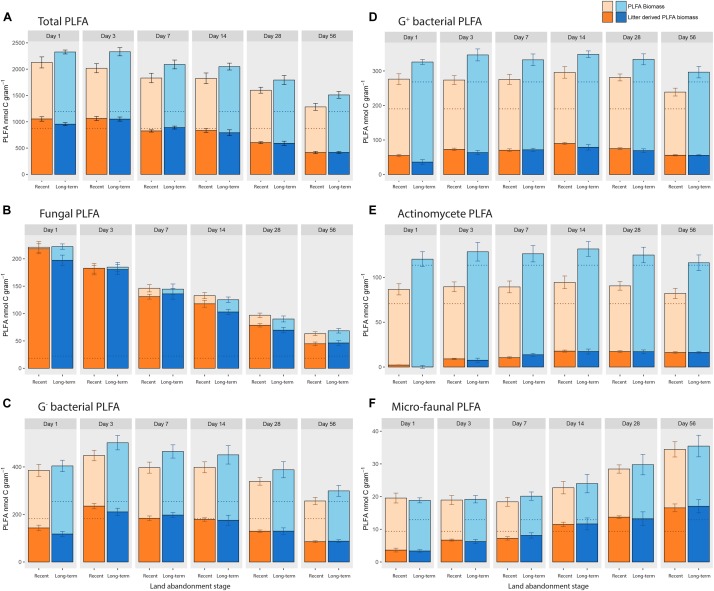
Means (± SE) PLFA biomass for different groups of PLFA biomarkers at respective sampling times, where the amount of litter-derived biomass is highlighted (± SE). Panel **(A)** represents the total amount of PLFA biomarkers found, whereby the remaining panels show specific biomass and labeling patterns for specific PLFA biomarkers that were appointed to the microbial groups **(B)** fungi, **(C)** G^−^ bacteria, **(D)** G^+^ bacteria, **(E)** actinomycetes, and **(F)** microfauna. Dotted lines show the corresponding control PLFA biomass without litter addition.

One day after the addition of ^13^C-labeled litter, the total amount of PLFA in both recent and long-term abandoned soils increased from approximately 1000 to more than 2000 nmol C per gram soil. The newly build up PLFA biomass corresponds to the amount of PLFA biomass that was litter-derived (Figure 2A). After day 3, the total amount of PLFA biomass gradually decreased, so that at day 56 the amount of PLFA biomass was still slightly increased compared to the situation without litter addition. Overall, there was only a marginally significant higher biomass in long-term abandoned soils compared to recently abandoned soils (χ^2^ = 2.814, *p* = 0.093). The higher microbial biomass in long-term abandoned soils was mainly due to a greater increase in bacterial biomass in response to litter addition, reflected by a significantly higher fungal/bacterial ratio for recently abandoned fields (0.186 ± 0.009) compared to long-term abandoned fields (0.158 ± 0.008) over the course of the experiment (χ^2^ = 4.2319, *p* = 0.039). No significant differences in the absolute amounts of litter-derived PLFA biomass (χ^2^ = 0.2295, *p* = 0.632) were found between land abandonment stages. However, when comparing the relative amounts of litter-derived PLFA biomass (as a percentage of PLFA biomass) between recent and long-term abandoned soils, there was a significant interaction effect between land abandonment stage and time (stage^∗^time: χ^2^ = 11.9256, *p* = 0.036; Table 4), with a relatively higher percentage of litter-derived PLFA in recently abandoned soils. Similar patterns were observed when examining individual PLFA biomarkers, where only a few biomarkers were significantly affected by land abandonment stage during the incubation experiment when testing for PLFA biomass (Supplementary Table S2) and the absolute amount of litter-derived PLFA biomass (Supplementary Table S3). The relative amounts of litter-derived PLFA biomass was significantly affected by land abandonment stage for nine out of the 24 individual PLFA biomarkers analyzed (Supplementary Table S4).

**FIGURE 3:**
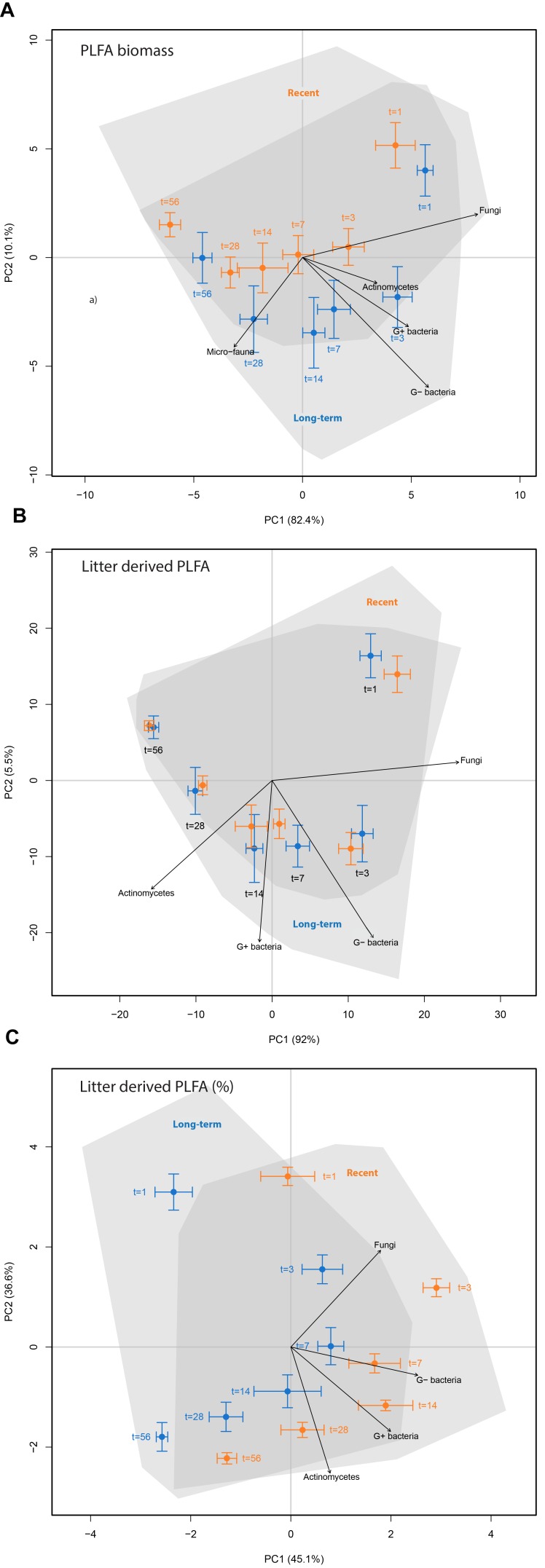
Principal component analyses of phospholipid fatty acids (PLFA) biomarkers to characterize soil microbial community structure in ex-arable soils (recent abandonment, orange vs. long-term abandonment, blue) and at different time points after ^13^C-labeled litter addition. PCA **(A)** is based on the relative abundance of 20 different PLFA biomarkers. PCA **(B)** is based on the absolute amount of each PLFA biomarker that was litter-derived. PCA **(C)** is based on the relative amount of each PLFA biomarker that was litter-derived. Arrows show indicative microbial groups (*r*^2^ with *p* < 0.05).

To visualize the effects of land abandonment stage and time on PLFA biomass and the absolute and relative amount of litter-derived PLFA, the information for all 24 PLFA biomarkers was combined in a principal component analysis (PCA) based on PLFA biomass (Figure 3A), the absolute amount of litter-derived PLFA (Figure 3B) and the relative amount of litter-derived PLFA biomass (Figure 3C). These analyses reveal a strong time effect during the incubation experiment for all three microbial community properties, whereby only the relative amount of litter-derived PLFA was clearly affected by land-abandonment stage (Figure 3C). The PCA revealed that some microbial groups change with time, with fungal PLFA being relatively abundant and enriched at the start of the experiment (Figure 3), while the enrichment of G^+^ bacteria and actinomycetes were indicative for later stages of ^13^C-litter decomposition (Figure 3B,C).

The microbial community properties (PLFA biomass, absolute and relative amount of litter-derived PLFA) are shown with respect to specific microbial groups: fungal PLFA (Figure 2B), G^−^ bacterial PLFA (Figure 2C), G^+^ bacterial PLFA (Figure 2D), actinomycete PLFA (Figure 2E), and micro-faunal PLFA (Figure 2F). For PLFA biomass, there was only a significant higher amount of PLFA biomarkers for G^+^ (χ^2^ = 4.612, *p* = 0.032) and actinomycete PLFA biomarkers (χ^2^ = 3.869, *p* = 0.052) in long-term abandoned soils. There were no significant effects of land abandonment stage on the absolute amount of litter-derived PLFA biomass for any of the microbial groups. When testing the fungal/bacterial ratio of excess ^13^C there was no effect of land abandonment stage (χ^2^ = 0.0176, *p* = 0.894), however, there was a significant interaction effect (time^∗^stage: χ^2^ = 11.474, *p* = 0.043). Thus, for most microbial groups there was a (marginally) significant interaction effect (time^∗^stage) when looking at the percentage of litter-derived PLFA biomass (Table 4), with only the fungal PLFA showing no detectable effect of land abandonment stage.

**Table 4:** Relative amount PLFA biomarker abundance for specific microbial groups that was litter-derived (%).

		Time (days)									
	*Land abandonment stage*	*1*	*3*	*7*	*14*	*28*	*56*		*χ^2^*	*p*	
Total PLFA	Recent	50.02	52.85	45.72	46.31	38.13	32.52	Stage:	5.759	0.016	^∗^
	Long-term	41.07	45.22	42.78	38.45	32.87	27.82	Stage^∗^Time:	11.926	0.036	^∗^
Fungal PLFA	Recent	97.79	101.03	89.17	87.62	80.60	70.38	Stage:	2.056	0.152	
	Long-term	87.83	96.83	91.82	81.31	76.20	66.78	Stage^∗^Time:	7.450	0.189	
G^−^ bacterial PLFA	Recent	37.15	52.21	46.13	45.02	38.13	33.87	Stage:	7.425	0.006	^∗∗^
	Long-term	28.37	41.42	41.88	37.64	33.05	29.69	Stage^∗^Time:	9.569	0.088	.
G^+^ bacterial PLFA	Recent	19.42	26.56	25.33	30.27	26.32	23.77	Stage:	2.434	0.119	
	Long-term	11.25	18.44	21.67	22.56	21.01	19.16	Stage^∗^Time:	14.569	0.012	^∗^
Actinomycete PLFA	Recent	1.95	10.53	12.02	18.73	18.80	20.29	Stage:	0.578	0.447	
	Long-term	0.65	6.47	12.03	13.91	15.29	15.39	Stage^∗^Time:	25.612	<0.001	^∗∗∗^
Micro-fauna PLFA	Recent	17.42	17.51	30.67	51.18	43.98	52.95	Stage:	0.574	0.449	
	Long-term	17.80	29.24	30.71	46.29	46.00	37.83	Stage^∗^Time:	13.016	0.023	^∗^

*Time indicates the total time after ^13^C-labeled litter addition for both recent and long-term abandoned soils. χ^2^ and p-values are the result of linear-mixed modeling (^∗∗∗^p < 0.001, ^∗∗^p < 0.01, ^∗^p < 0.05, ^.^p < 0.1).*

### Succession of Soil Microbial Groups During Litter Decomposition

When examining the build-up of litter-derived PLFA biomass in Figure 2, we found that each microbial group had its own distinct substrate derived ^13^C incorporation pattern over time. This observation is apparent in Figure 3 where indicative microbial groups change over time during litter decomposition. This is also shown when looking at the ^13^C excess patterns over time for individual PLFA biomarkers, with distinct patterns for different PLFA biomarkers (Supplementary Figure S2). To compare these ^13^C incorporation patterns between microbial groups, the amount of excess ^13^C was normalized for each microbial group, and all group-specific temporal patterns were subsequently plotted together for recent (Supplementary Figure S1a) and long-term abandoned soils (Supplementary Figure S1b). Supplementary Figure S1 shows that fungi have their highest amount of ^13^C incorporation already 1 day after the addition of ^13^C-labeled litter, with a steep decrease in the amount of incorporated ^13^C directly afterward. G^−^ bacteria also accumulate ^13^C rapidly, but reach their peak in ^13^C incorporation at day 3. G^+^ bacteria and related actinomycetes both reach their maximum level of litter-derived ^13^C incorporation at day 14. However, after this peak, the amount of excess ^13^C remained relatively stable in actinomycetes in contrast to the rapid decrease observed for ^13^C in G^+^ bacteria. The PLFA biomarker specific to micro-fauna reaches its maximum amount of ^13^C incorporation only after 28 days, after which it remains generally stable. When testing the normalized amounts of ^13^C excess, there is a significant interaction effect between microbial group and time (time^∗^microbial_group: χ^2^ = 207.845, *p* < 0.001), confirming distinct ^13^C incorporation patterns between microbial groups. The ^13^C incorporation patterns found for microbial groups over time are unaffected by land abandonment stage (stage^∗^time^∗^microbial_group; χ^2^ = 20.702, *p* = 0.415).

## Discussion

In this study, we sought to examine how soil microbial communities and soil microbial C flows in ex-arable soils respond to litter addition and subsequent litter decomposition in both recent and long-term abandoned soils, with the ultimate goal of providing quantitative litter-derived C flows through the soil microbial community in response to a common form of land-use change. Litter-derived C flows followed a clear succession of microbial groups in the detrital microbial food web (fungi > G^−^ bacteria > G^+^ bacteria ≥ actinomycetes > micro-fauna). This pattern was remarkably comparable across all soils, regardless of land abandonment stage. Furthermore, we also observed that in terms of absolute energy flows there were no differences between microbial communities of different ex-arable soils. However, microbial communities showed a higher uptake efficiency of litter-derived C in recently abandoned soils as compared to long-term abandoned soils.

### Influence of Litter Addition and Land-Abandonment Stage on the Soil Microbial Community

We hypothesized that long-term abandoned soils would have a higher capacity for decomposition of added litter, as compared to recently abandoned soils. Our results show that long-term abandoned soils had an initial higher microbial biomass and that this difference persisted after litter addition. This might have caused a higher (litter-derived) C mineralization rate after litter addition in long-term than recently abandoned soils. Even though microbial parameters are often not statistically different between secondary states, which might be caused by the low number of replicates, there is a clear trend toward higher soil microbial biomass and activity in long-term abandoned soils. This is in accordance with the research of Holtkamp et al. (2011) in the same study area, who show an increase in C mineralization by the soil community following land-abandonment, as well as an increase in soil microbial biomass. The higher soil microbial biomass in long-term abandoned soils was mainly caused by an increase in G^+^ bacteria and actinomycetes in long-term abandoned soils, both before and after litter addition. A possible explanation for the higher abundance of these microbial groups in long-term abandoned soils, is the larger fraction of recalcitrant SOM in the C pool of long-term abandoned soils as opposed to recently abandoned soils (Kramer and Gleixner, 2006, 2008; Holtkamp et al., 2008).

The fungal community showed a large increase in biomass in response to litter addition, regardless of land abandonment stage. This response may be related to the fact that, via their use of exploratory hyphae, fungi can rapidly access added litter and transport soil inorganic N to stimulate the production of fungal biomass (Frey et al., 2000, 2003). In this way, they can play an important quantitative role in litter decomposition. All other microbial groups examined also showed an increase in biomass after litter addition, although effects were less pronounced compared to the fungal response. G^+^ bacteria and actinomycetes retain a higher biomass after litter addition in long-term abandoned soils, whereby initial differences between ex-arable soils in terms of microbial community structure are still detectable. This suggests that, while litter addition caused a total increase of soil microbial community biomass and fungal/bacterial ratio, the initial differences in soil microbial community structure between the two types of ex-arable soils are not affected by litter addition. It could be possible that important community shifts occurred within the broad microbial groups represented by the analyzed PLFA biomarkers, such as recently shown within the fungal community (Morriën et al., 2017). It would therefore be of interest in future studies to analyze community dynamics during the flow of “brown” C in soil from different land abandonment stages at a higher level of functional and taxonomic detail.

### Litter-C Uptake (Efficiency) of Soil Microbes Following Land Abandonment

We hypothesized that litter decomposition would be higher in long-term abandoned fields due to an increased activity and efficiency of the soil fungal community. Fungi have long been considered to be the main responsible microbial group to decomposing labile and recalcitrant SOM in soil food web ecology (de Boer et al., 2005; Holtkamp et al., 2008). However, we did not find a build-up of fungal biomass or a community shift toward fungal dominated microbial communities in long-term abandoned soils, which is in line with other recent studies (Creamer et al., 2016; Morriën et al., 2017). In addition, we also did not observe an increased activity or efficiency of the fungal community in long-term abandoned soils when comparing the absolute and relative amounts of litter-derived C uptake. This is in contrast to what was previously found by Morriën et al. (2017), who traced C flows entering the soil system via the green pathway by ^13^C pulse-labeling. Our results showed a lower litter-C uptake efficiency of the microbial community in long-term abandoned soils (Table 4). The counteracting trends of increasing bacterial biomass following land-abandonment, together with a decreased bacterial C uptake efficiency, resulted in a similar net C flow for bacterial communities in both recent and long-term abandoned soils. These results are somewhat in contrast with the recent view that increased C uptake efficiency by the soil food web in long-term ex-arable fields is due to a tightening of soil food webs (Morriën et al., 2017). It seems that long-term land abandonment has a positive influence on rhizosphere C uptake efficiency, but not on detritus-derived C uptake efficiency. This is in agreement with previous observations for the transition from grassland to woodlands, where it was suggested that soil microbes in long-term abandoned soils appear to be less efficient in converting organic matter C to microbial biomass (McCulley et al., 2004; Liao and Boutton, 2008). A possible explanation is that root C biomass is relatively greater than the amount of C in SOM pools in long-term abandoned fields, which causes a shift from SOM-derived C toward root-derived C input in the soil microbial food web as reflected in the efficiency of the microbial community in processing these C flows.

### Litter-Derived C Incorporation in the Soil Microbial Food Web

A few remarks are appropriate when interpreting litter-derived ^13^C incorporation patterns in the soil microbial food web. First, it should be noted that the different fractions of plant litter may have contained different levels of ^13^C enrichment. This might have affected the labeling of the microbial groups within our incubation experiment. If the consumed litter would have a higher ^13^C enrichment compared to the overall litter, the amount of litter-derived C in the microbial community would have been overestimated. However, from our results it seems that this is not the case since the litter-derived biomass corresponds well to the build-up in microbial biomass after litter addition (Figure 2). Future experiments in which different fractions of the organic amendment are separately labeled would provide additional insight in the timing and quantity of microbial activities in relation to specific organic matter fractions. Our study also used a relative short time frame of 8 weeks with an estimated litter decomposition around 15–20% (unpublished data from litter bag experiment, in accordance with results of Cornelissen and Thompson, 1997). Therefore, it is also possible that much of the litter-derived C in this study came from the easy decomposable parts of plant remains, and that truly recalcitrant substrate decomposition was not considered in our experimental design.

Interestingly, each soil microbial group examined in our study found its own “temporal window” in terms of having a maximum amount of litter-derived C incorporation (Figure 3B and Supplementary Figure S1), giving more insight into the structure of the soil microbial detrital food web. Overall, there was a clear succession of microbial groups incorporating litter-derived C in their biomass both in time (1–56 days) and quantity (high-low), in the order of fungi > G^−^ bacteria > G^+^ bacteria ≥ actinomycetes > micro-fauna. With respect to the bacteria, our findings support earlier findings that G^−^ bacteria are more closely associated with the initial decomposition of easily decomposable materials, while G^+^ bacteria (and related actinomycetes) are later involved in the decomposition of more recalcitrant organic matter (Kramer and Gleixner, 2006, 2008). The fast and high decomposition activity of the saprotrophic fungal community has recently been described for the root-derived C flow in soils (e.g., Ballhausen and de Boer, 2016; Pausch et al., 2016), and this patterns also appears to hold for litter-derived C flows. This results in a large build-up of fungal biomass after application of litter, which then again rapidly decreases over time. While fungi have a net loss of litter-derived C directly afterward, bacterial groups are still building-up their biomass on litter-derived C inputs up to 14 days after litter addition. A possible explanation could be that fungal hyphae are a C source for the bacterial community (Ballhausen and de Boer, 2016). Remarkably, soil microbial ^13^C-incorporation patterns were very similar across all the fields examined, both in terms of the timing and absolute values of C incorporation. These similarities suggest that the observed soil microbial detrital food web structure is relatively stable over a range of biotic and abiotic conditions. It would be necessary to examine such soil microbial food web dynamics across a wider range of soil systems before this can be generalized further.

## Conclusion

This study gives a first quantitative insight in how litter-derived C flows through the detritus-based soil microbial food web in soils from different stages of land abandonment. By following litter-derived ^13^C incorporation patterns in the microbial community, we found that there is a clear succession of microbial groups, irrespective of land abandonment stage, during litter decomposition (fungi > G^−^ bacteria > G^+^ bacteria ≥ actinomycetes > micro-fauna). The decreased efficiency of the soil microbial community to transform SOM-derived C pools suggests that following land abandonment, a shift occurs from SOM-derived to root-derived based soil food webs, caused by a relative change in the importance of C input sources following land abandonment. Information of quantitative C flows through soil microbial groups can help to improve existing soil food web models, by giving structure to the “detritus black box,” which currently lumps all microbial groups and their inherent traits. This perspective may lead to additional insights regarding the role and importance of the diverse microbial community in ecosystem processes like C and N mineralization, with the ultimate aim to improve predictions of ecosystem structure and important ecosystem services like C sequestration.

## Author Contributions

AH, PdR, PB, and GK conceived and planned the experiments. AH carried out the experiments and wrote the manuscript with the support of PdR, PB, and GK.

## Conflict of Interest Statement

The authors declare that the research was conducted in the absence of any commercial or financial relationships that could be construed as a potential conflict of interest.
