# Supplementary material for: Modulation of Litter Decomposition by the Soil Microbial Food Web Under Influence of Land Use Change
Source: Front Microbiol. 2018 Nov 26;9:2860. doi: 10.3389/fmicb.2018.02860 (PMC6275175; doi:10.3389/fmicb.2018.02860)
Supplement: Supplementary file 1 [file Data_Sheet_1.docx]

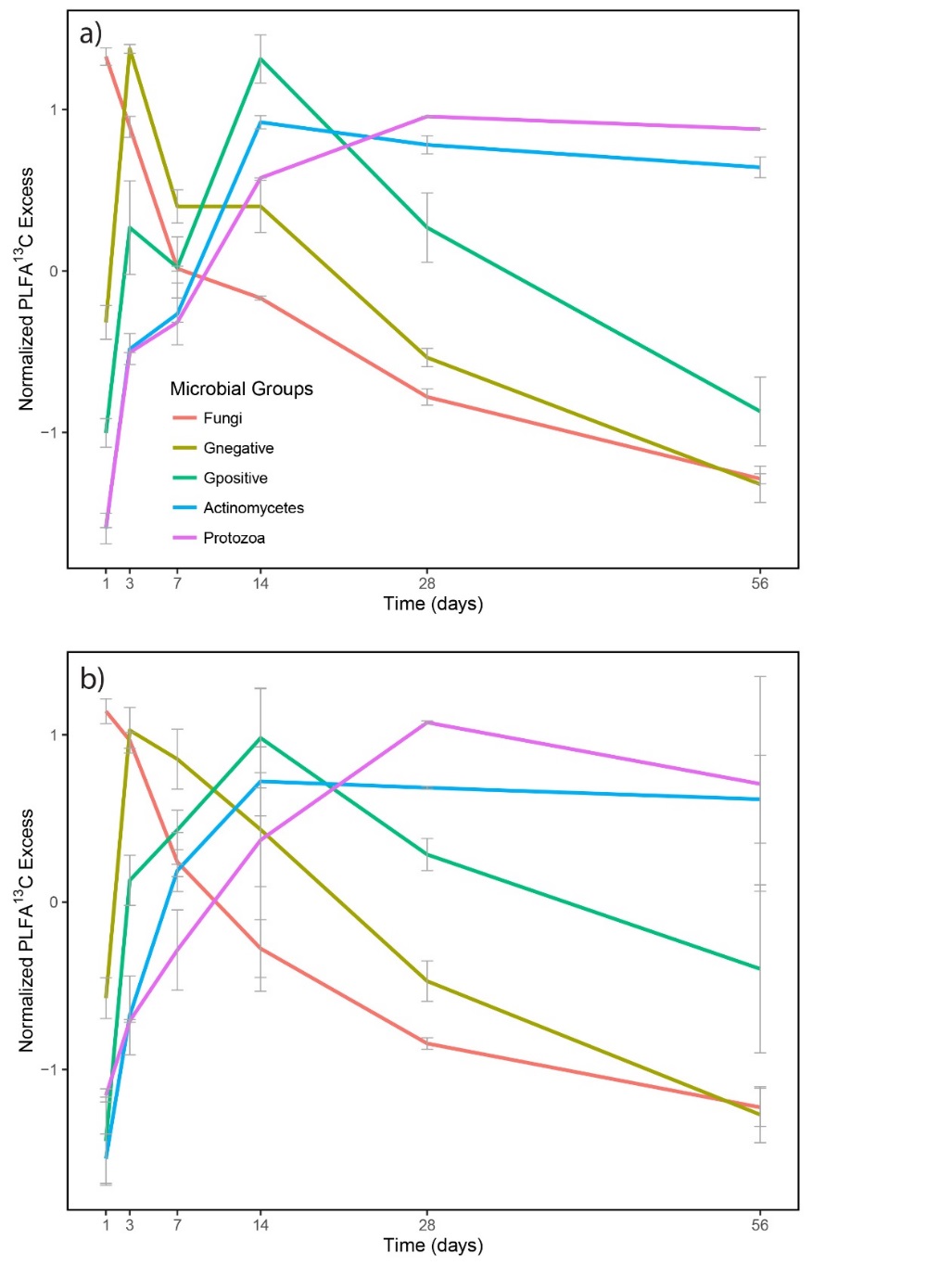


**Figure S1** | Mean normalized amounts of ^13^C excess (± SE) in different present microbial groups as measured by the amount of ^13^C incorporated in PLFA biomarkers over time for a) recent and b) long-term abandoned soils **.**

**
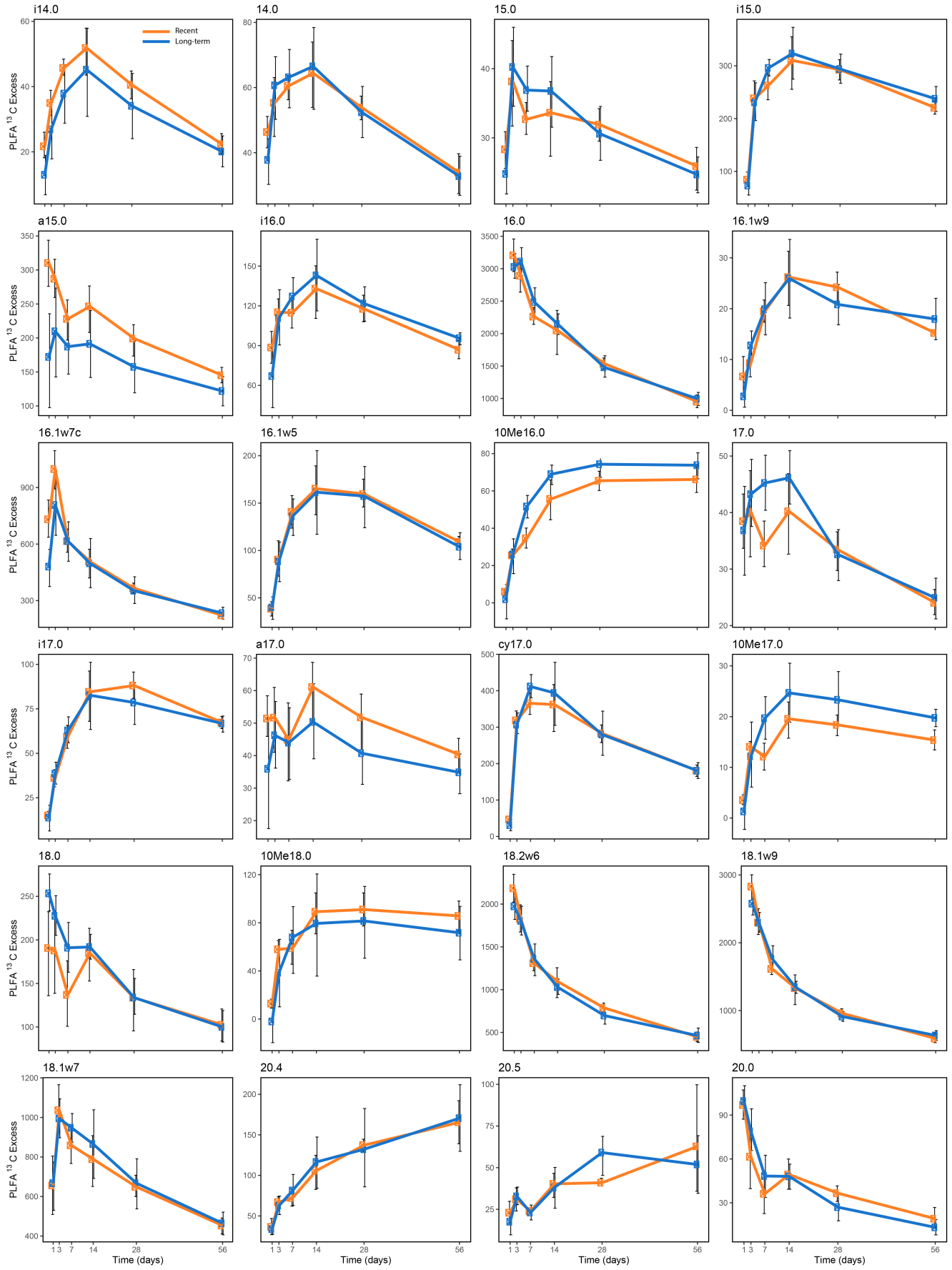
**

**Figure S2 |** The amount of excess ^13^C in different PLFA biomarkers over time since ^13^C-labelled litter addition. Lines represent means of recently abandoned soils (orange) and long-term abandoned soils (blue) ± SE.

**Table S1** | Soil microbial community abundances for specific PLFA biomarkers (nmol g soil^-1^, mean ± SE) in recent and long-term abandoned soils. χ^2^ and p-values are the result of linear-mixed modelling (*** p<0.001, ** p<0.01, * p<0.05, ˙ p<0.1).

|  |  |  |  |  |  |
| --- | --- | --- | --- | --- | --- |
|  | **Land abandonment stage** | |  |  |  |
| ***PLFA marker*** | ***Recent*** | ***Long-term*** | ***χ^2^*** | ***p*** |  |
| i14.0 | 6.690 | 7.912 | 1.1172 | 0.291 |  |
| 14.0 | 13.405 | 16.112 | 0.577 | 0.448 |  |
| 15.0 | 8.877 | 11.124 | 0.8603 | 0.354 |  |
| i15.0 | 80.546 | 110.960 | 3.6654 | 0.056 | **.** |
| a15.0 | 45.174 | 53.812 | 7.7192 | **0.005** | ****** |
| i16.0 | 29.844 | 49.224 | 8.2789 | **0.004** | ****** |
| 16.0 | 193.321 | 248.907 | 1.1057 | 0.293 |  |
| 16.1ω9 | 6.796 | 8.686 | 4.519 | **0.034** | ***** |
| 16.1ω7c | 60.891 | 81.662 | 1.2551 | 0.263 |  |
| 16.1ω5 | 40.462 | 49.692 | 2.6685 | 0.102 |  |
| 10Me16.0 | 37.465 | 58.984 | 10.204 | **0.001** | ****** |
| 17.0 | 6.237 | 8.203 | 3.6888 | 0.055 | **.** |
| i17.0 | 20.195 | 28.513 | 4.0489 | **0.044** | ***** |
| a17.0 | 14.476 | 19.076 | 9.1756 | **0.002** | ****** |
| cy17.0 | 28.628 | 37.282 | 4.048 | **0.044** | ***** |
| 10Me17.0 | 8.446 | 15.643 | 3.9016 | **0.048** | ***** |
| 18.0 | 35.134 | 44.516 | 1.2908 | 0.256 |  |
| 10Me18.0 | 24.804 | 35.528 | 10.798 | 0.001 |  |
| 18.2ω6 | 18.289 | 22.084 | 2.9288 | 0.087 | **.** |
| 18.1ω9 | 76.986 | 103.919 | 0.8014 | 0.371 |  |
| 18.1ω7 | 92.886 | 135.992 | 8.8574 | **0.003** | ****** |
| 20.4 | 9.374 | 13.032 | 1.1302 | 0.288 |  |
| 20.5 | 3.111 | 3.749 | 2.8009 | 0.094 | . |
| 20.0 | 10.301 | 12.750 | 0.232 | 0.630 |  |

**Table S2 |** Mean abundance of specific PLFA biomarkers (nmol g soil^-1^) over time after ^13^C-labelled litter addition for both recent and log-term abandoned soils. χ^2^ and p-values are the result of linear-mixed modelling (*** p<0.001, ** p<0.01, * p<0.05, ˙ p<0.1).

|  |  | **Time (days)** | | | | | |  | |  |  |  |  |
| --- | --- | --- | --- | --- | --- | --- | --- | --- | --- | --- | --- | --- | --- |
| ***PLFA marker*** | ***Land abandonment stage*** | ***1*** | ***3*** | ***7*** | ***14*** | ***28*** | ***56*** |  | ***χ^2^*** | | ***p*** |  | |
| i14.0 | Recent | 10.20 | 10.90 | 12.68 | 13.80 | 12.32 | 8.42 | Stage: | 0.052 | | 0.820 |  | |
|  | Long-term | 10.79 | 11.97 | 12.38 | 13.53 | 12.42 | 9.20 | Stage*Time: | 0.027 | | 0.869 |  | |
| 14.0 | Recent | 19.15 | 19.78 | 20.83 | 21.62 | 19.97 | 16.46 | Stage: | 6.023 | | **0.014** | * | |
|  | Long-term | 22.33 | 24.26 | 23.17 | 24.73 | 22.90 | 19.84 | Stage*Time: | 0.008 | | 0.928 |  | |
| 15.0 | Recent | 13.94 | 14.48 | 14.35 | 13.96 | 13.84 | 13.06 | Stage: | 5.540 | | **0.019** | * | |
|  | Long-term | 16.51 | 16.98 | 16.06 | 15.93 | 15.45 | 14.99 | Stage*Time: | 0.361 | | 0.548 |  | |
| i15.0 | Recent | 98.48 | 104.93 | 109.28 | 119.08 | 115.26 | 98.02 | Stage: | 3.926 | | **0.048** | * | |
|  | Long-term | 129.76 | 143.38 | 139.64 | 147.77 | 142.81 | 126.43 | Stage*Time: | 0.477 | | 0.490 |  | |
| a15.0 | Recent | 86.42 | 78.14 | 74.26 | 76.08 | 68.88 | 57.11 | Stage: | 0.027 | | 0.870 |  | |
|  | Long-term | 78.60 | 78.99 | 71.20 | 72.98 | 68.93 | 59.38 | Stage*Time: | 1.778 | | 0.182 |  | |
| i16.0 | Recent | 45.32 | 44.77 | 44.23 | 47.48 | 43.70 | 36.59 | Stage: | 4.062 | | **0.044** | * | |
|  | Long-term | 63.72 | 67.51 | 65.45 | 67.50 | 62.20 | 55.61 | Stage*Time: | 0.180 | | 0.672 |  | |
| 16.0 | Recent | 543.54 | 495.10 | 449.03 | 437.31 | 373.71 | 296.02 | Stage: | 2.471 | | 0.116 |  | |
|  | Long-term | 592.72 | 566.09 | 490.09 | 478.87 | 401.78 | 338.12 | Stage*Time: | 0.338 | | 0.561 |  | |
| 16.1ω9 | Recent | 8.25 | 8.74 | 9.60 | 10.48 | 10.36 | 8.11 | Stage: | 0.715 | | 0.398 |  | |
|  | Long-term | 9.84 | 9.79 | 11.53 | 11.84 | 11.75 | 9.04 | Stage*Time: | 0.337 | | 0.562 |  | |
| 16.1ω7c | Recent | 160.91 | 176.55 | 136.34 | 128.67 | 106.46 | 77.62 | Stage: | 0.413 | | 0.521 |  | |
|  | Long-term | 156.98 | 185.38 | 156.24 | 143.18 | 120.64 | 91.34 | Stage*Time: | 3.639 | | 0.056 |  | |
| 16.1ω5 | Recent | 50.70 | 51.33 | 55.11 | 64.13 | 66.12 | 50.26 | Stage: | 0.287 | | 0.592 |  | |
|  | Long-term | 55.82 | 59.61 | 60.54 | 67.81 | 69.06 | 51.48 | Stage*Time: | 2.406 | | 0.121 |  | |
| 10Me16.0 | Recent | 43.60 | 43.32 | 42.29 | 45.29 | 44.95 | 41.74 | Stage: | 2.958 | | 0.085 |  | |
|  | Long-term | 62.78 | 65.56 | 64.25 | 67.37 | 65.61 | 61.51 | Stage*Time: | 0.160 | | 0.689 |  | |
| 17.0 | Recent | 13.23 | 12.94 | 12.45 | 12.45 | 11.52 | 9.87 | Stage: | 2.785 | | 0.095 |  | |
|  | Long-term | 15.28 | 15.04 | 14.17 | 14.52 | 12.32 | 11.84 | Stage*Time: | 0.104 | | 0.747 |  | |
| i17.0 | Recent | 23.41 | 23.92 | 26.62 | 30.73 | 31.33 | 28.24 | Stage: | 4.296 | | **0.038** | * | |
|  | Long-term | 29.33 | 31.67 | 32.59 | 35.55 | 36.35 | 34.45 | Stage*Time: | 0.717 | | 0.397 |  | |
| a17.0 | Recent | 22.70 | 21.96 | 21.31 | 22.52 | 22.03 | 18.92 | Stage: | 1.165 | | 0.280 |  | |
|  | Long-term | 24.57 | 24.94 | 23.36 | 24.47 | 23.46 | 20.78 | Stage*Time: | 0.207 | | 0.649 |  | |
| cy17.0 | Recent | 35.89 | 62.12 | 70.23 | 74.28 | 63.51 | 47.50 | Stage: | 0.913 | | 0.339 |  | |
|  | Long-term | 38.62 | 69.12 | 79.72 | 81.92 | 68.91 | 53.37 | Stage*Time: | 0.003 | | 0.960 |  | |
| 10Me17.0 | Recent | 11.40 | 11.31 | 11.30 | 11.78 | 10.63 | 9.45 | Stage: | 3.215 | | 0.073 |  | |
|  | Long-term | 18.77 | 19.83 | 19.19 | 20.18 | 17.61 | 16.83 | Stage*Time: | 0.365 | | 0.546 |  | |
| 18.0 | Recent | 62.43 | 56.84 | 54.97 | 57.07 | 53.60 | 47.33 | Stage: | 0.868 | | 0.090 |  | |
|  | Long-term | 71.34 | 68.03 | 62.23 | 64.39 | 58.34 | 53.76 | Stage*Time: | 1.267 | | 0.260 |  | |
| 10Me18.0 | Recent | 31.69 | 34.93 | 35.81 | 37.42 | 34.98 | 30.99 | Stage: | 1.694 | | 0.193 |  | |
|  | Long-term | 38.85 | 43.25 | 42.94 | 44.22 | 41.78 | 38.00 | Stage*Time: | 0.026 | | 0.871 |  | |
| 18.2ω6 | Recent | 221.31 | 179.33 | 146.25 | 132.80 | 96.94 | 63.21 | Stage: | 0.002 | | 0.961 |  | |
|  | Long-term | 222.38 | 184.92 | 144.64 | 125.27 | 90.09 | 68.51 | Stage*Time: | 0.012 | | 0.911 |  | |
| 18.1ω9 | Recent | 388.05 | 311.50 | 252.08 | 225.87 | 179.44 | 135.99 | Stage: | 1.349 | | 0.245 |  | |
|  | Long-term | 408.05 | 348.18 | 284.91 | 251.59 | 199.08 | 161.72 | Stage*Time: | 0.088 | | 0.767 |  | |
| 18.1ω7 | Recent | 188.85 | 209.45 | 190.55 | 195.55 | 169.43 | 131.56 | Stage: | 0.653 | | 0.419 |  | |
|  | Long-term | 208.72 | 247.40 | 229.23 | 225.82 | 198.64 | 154.59 | Stage*Time: | 0.452 | | 0.501 |  | |
| 20.4 | Recent | 19.56 | 18.95 | 18.42 | 22.72 | 28.43 | 34.45 | Stage: | 0.019 | | 0.891 |  | |
|  | Long-term | 18.85 | 19.19 | 20.14 | 23.97 | 29.78 | 35.46 | Stage*Time: | 0.038 | | 0.846 |  | |
| 20.5 | Recent | 8.45 | 7.80 | 7.14 | 8.25 | 8.13 | 9.53 | Stage: | 0.329 | | 0.566 |  | |
|  | Long-term | 7.14 | 6.84 | 6.61 | 7.13 | 7.61 | 9.38 | Stage*Time: | 0.058 | | 0.810 |  | |
| 20.0 | Recent | 20.90 | 18.66 | 16.90 | 16.24 | 14.36 | 12.10 | Stage: | 7.262 | | **0.007** | ** | |
|  | Long-term | 24.97 | 22.59 | 19.30 | 19.01 | 16.48 | 14.85 | Stage*Time: | 0.609 | | 0.435 |  | |

**Table S3 –** Mean ^13^C excess (pmol g soil^-1^) of specific PLFA biomarkers over time after ^13^C-labelled litter addition for both recent and long-term abandoned soils. χ^2^ and p-values are the result of linear-mixed modelling (*** p<0.001, ** p<0.01, * p<0.05, ˙ p<0.1).

|  |  | **Time (days)** | | | | | |  |  |  |  |
| --- | --- | --- | --- | --- | --- | --- | --- | --- | --- | --- | --- |
| ***PLFA marker*** | ***Land abandonment stage*** | ***1*** | ***3*** | ***7*** | ***14*** | ***28*** | ***56*** |  | ***χ^2^*** | ***p*** |  |
| i14.0 | Recent | 21.7 | 35.0 | 45.7 | 55.3 | 40.5 | 22.3 | Stage: | 7.332 | 0.392 |  |
|  | Long-term | 13.0 | 26.9 | 37.8 | 45.2 | 34.1 | 20.1 | Stage*Time: | 0.357 | 0.550 |  |
| 14.0 | Recent | 46.3 | 55.3 | 60.5 | 69.0 | 53.8 | 33.9 | Stage: | 0.014 | 0.905 |  |
|  | Long-term | 37.8 | 60.7 | 63.1 | 66.5 | 52.4 | 32.8 | Stage*Time: | 0.002 | 0.97 |  |
| 15.0 | Recent | 28.3 | 38.1 | 32.7 | 36.1 | 31.9 | 25.9 | Stage: | 0.006 | 0.936 |  |
|  | Long-term | 24.8 | 40.2 | 36.9 | 36.8 | 30.6 | 24.7 | Stage*Time: | 0.285 | 0.593 |  |
| i15.0 | Recent | 84.3 | 238.3 | 263.0 | 334.1 | 292.6 | 220.5 | Stage: | 0.059 | 0.809 |  |
|  | Long-term | 73.1 | 230.7 | 295.9 | 323.4 | 293.8 | 237.5 | Stage*Time: | 0.100 | 0.752 |  |
| a15.0 | Recent | 310.3 | 287.0 | 228.0 | 264.4 | 199.2 | 145.0 | Stage: | 1.767 | 0.184 |  |
|  | Long-term | 172.1 | 209.8 | 187.3 | 191.3 | 157.5 | 121.9 | Stage*Time: | 11.486 | **<0.001** | ******* |
| i16.0 | Recent | 88.6 | 115.2 | 114.8 | 143.5 | 117.7 | 86.8 | Stage: | 0.001 | 0.975 |  |
|  | Long-term | 67.0 | 110.7 | 127.0 | 143.0 | 121.7 | 95.6 | Stage*Time: | 1.219 | 0.270 |  |
| 16.0 | Recent | 3206.8 | 2891.2 | 2260.8 | 2194.1 | 1537.1 | 950.0 | Stage: | 0.207 | 0.650 |  |
|  | Long-term | 3027.4 | 3109.7 | 2486.9 | 2147.7 | 1479.6 | 994.2 | Stage*Time: | 0.031 | 0.860 |  |
| 16.1ω9 | Recent | 6.6 | 9.3 | 19.4 | 28.1 | 24.2 | 15.2 | Stage: | 0.537 | 0.464 |  |
|  | Long-term | 2.7 | 12.7 | 19.9 | 25.9 | 20.8 | 18.0 | Stage*Time: | 1.355 | 0.244 |  |
| 16.1ω7c | Recent | 730.9 | 997.7 | 615.3 | 546.6 | 365.1 | 221.7 | Stage: | 0.548 | 0.459 |  |
|  | Long-term | 480.3 | 807.1 | 616.4 | 497.8 | 352.5 | 233.5 | Stage*Time: | 6.999 | **0.008** | ****** |
| 16.1ω5 | Recent | 38.6 | 90.4 | 140.7 | 178.2 | 159.6 | 109.1 | Stage: | 0.072 | 0.788 |  |
|  | Long-term | 39.9 | 88.5 | 136.1 | 161.5 | 157.4 | 103.9 | Stage*Time: | 0.010 | 0.922 |  |
| 10Me16.0 | Recent | 5.9 | 25.4 | 34.6 | 60.3 | 65.5 | 66.1 | Stage: | 2.820 | 0.093 | . |
|  | Long-term | 1.9 | 26.0 | 51.6 | 69.0 | 74.3 | 73.8 | Stage*Time: | 0.236 | 0.627 |  |
| 17.0 | Recent | 38.5 | 40.1 | 34.2 | 43.3 | 33.4 | 24.0 | Stage: | 0.268 | 0.605 |  |
|  | Long-term | 36.9 | 43.2 | 45.2 | 46.2 | 32.6 | 24.9 | Stage*Time: | 0.912 | 0.340 |  |
| i17.0 | Recent | 15.0 | 36.1 | 59.3 | 91.4 | 88.1 | 67.3 | Stage: | 0.010 | 0.921 |  |
|  | Long-term | 13.8 | 38.5 | 62.8 | 82.7 | 78.6 | 66.8 | Stage*Time: | NA | NA |  |
| a17.0 | Recent | 51.4 | 51.7 | 44.9 | 65.9 | 51.8 | 40.4 | Stage: | 0.651 | 0.420 |  |
|  | Long-term | 35.9 | 46.3 | 44.0 | 50.4 | 40.7 | 34.8 | Stage*Time: | 0.024 | 0.878 |  |
| cy17.0 | Recent | 46.7 | 318.6 | 365.3 | 391.7 | 283.0 | 180.3 | Stage: | 0.011 | 0.917 |  |
|  | Long-term | 31.0 | 306.8 | 411.5 | 393.9 | 279.8 | 180.3 | Stage*Time: | NA | NA |  |
| 10Me17.0 | Recent | 3.5 | 14.0 | 12.1 | 21.2 | 18.4 | 15.4 | Stage: | 0.961 | 0.327 |  |
|  | Long-term | 1.3 | 12.1 | 19.7 | 24.7 | 23.3 | 19.8 | Stage*Time: | 0.080 | 0.778 | . |
| 18.0 | Recent | 190.6 | 187.7 | 136.9 | 198.3 | 133.6 | 102.4 | Stage: | 3.679 | 0.055 |  |
|  | Long-term | 253.4 | 227.6 | 191.0 | 191.8 | 133.7 | 100.0 | Stage*Time: | 5.453 | **0.020** | ***** |
| 10Me18.0 | Recent | 12.8 | 58.0 | 58.9 | 96.3 | 91.0 | 85.7 | Stage: | 0.053 | 0.818 |  |
|  | Long-term | -2.0 | 38.4 | 67.8 | 79.5 | 81.5 | 71.7 | Stage*Time: | 0.740 | 0.390 |  |
| 18.2ω6 | Recent | 2183.7 | 1827.7 | 1308.4 | 1178.2 | 785.9 | 447.3 | Stage: | 0.568 | 0.451 |  |
|  | Long-term | 1973.6 | 1804.3 | 1359.8 | 1029.0 | 697.3 | 462.8 | Stage*Time: | 0.204 | 0.652 |  |
| 18.1ω9 | Recent | 2827.6 | 2296.8 | 1613.3 | 1424.8 | 958.7 | 586.4 | Stage: | 0.024 | 0.877 |  |
|  | Long-term | 2576.7 | 2295.3 | 1762.0 | 1344.0 | 912.3 | 632.1 | Stage*Time: | 0.365 | 0.546 |  |
| 18.1ω7 | Recent | 655.8 | 1036.2 | 858.1 | 850.5 | 648.9 | 452.9 | Stage: | 0.292 | 0.589 |  |
|  | Long-term | 667.3 | 994.9 | 947.6 | 865.4 | 667.6 | 464.4 | Stage*Time: | NA | NA |  |
| 20.4 | Recent | 36.4 | 67.0 | 72.2 | 114.9 | 137.3 | 165.6 | Stage: | 0.010 | 0.922 |  |
|  | Long-term | 33.7 | 62.5 | 81.3 | 116.5 | 132.2 | 170.5 | Stage*Time: | 0.172 | 0.678 |  |
| 20.5 | Recent | 22.9 | 31.0 | 23.9 | 43.6 | 41.0 | 62.8 | Stage: | 0.387 | 0.534 |  |
|  | Long-term | 17.5 | 32.8 | 23.1 | 38.1 | 59.0 | 51.9 | Stage*Time: | 0.069 | 0.793 |  |
| 20.0 | Recent | 96.9 | 61.4 | 36.1 | 52.6 | 36.5 | 19.1 | Stage: | 0.007 | **0.934** |  |
|  | Long-term | 99.6 | 78.9 | 48.3 | 48.1 | 27.0 | 13.2 | Stage*Time: | 1.284 | 0.257 |  |

**Table S4 –** Relative amount PLFA biomarker abundance that was litter-derived (%). Time indicates the total time after ^13^C-labelled litter addition for both recent and long-term abandoned soils. χ^2^ and p-values are the result of linear-mixed modelling (*** p<0.001, ** p<0.01, * p<0.05, ˙ p<0.1).

|  |  | **Time (days)** | | | | | |  |  |  |  |
| --- | --- | --- | --- | --- | --- | --- | --- | --- | --- | --- | --- |
| ***PLFA marker*** | ***Land abandonment stage*** | ***1*** | ***3*** | ***7*** | ***14*** | ***28*** | ***56*** |  | ***χ^2^*** | ***p*** |  |
| i14.0 | Recent | 20.80 | 30.97 | 36.21 | 39.93 | 32.58 | 26.84 | Stage: | 1.937 | 0.164 |  |
|  | Long-term | 11.10 | 21.04 | 28.69 | 31.02 | 26.95 | 22.42 | Stage*Time: | 1.611 | 0.204 |  |
| 14.0 | Recent | 23.67 | 27.41 | 29.53 | 31.60 | 26.91 | 20.93 | Stage: | 0.976 | 0.323 |  |
|  | Long-term | 17.03 | 25.01 | 26.74 | 26.91 | 23.69 | 17.16 | Stage*Time: | 0.153 | 0.70 |  |
| 15.0 | Recent | 19.57 | 26.30 | 23.24 | 24.72 | 23.01 | 20.35 | Stage: | 0.887 | 0.346 |  |
|  | Long-term | 15.13 | 23.53 | 22.59 | 23.06 | 20.75 | 16.89 | Stage*Time: | 0.090 | 0.765 |  |
| i15.0 | Recent | 8.04 | 22.57 | 23.97 | 27.95 | 25.14 | 22.85 | Stage: | 10.487 | **0.001** | ****** |
|  | Long-term | 6.13 | 16.52 | 21.53 | 22.19 | 21.02 | 19.03 | Stage*Time: | NA | NA |  |
| a15.0 | Recent | 35.54 | 36.78 | 30.49 | 34.95 | 28.66 | 25.63 | Stage: | 3.800 | 0.051 |  |
|  | Long-term | 19.26 | 24.78 | 25.25 | 24.97 | 22.24 | 20.37 | Stage*Time: | 12.396 | **<0.001** | ******* |
| i16.0 | Recent | 19.03 | 25.51 | 25.78 | 29.98 | 26.58 | 24.22 | Stage: | 1.976 | 0.160 |  |
|  | Long-term | 11.61 | 17.52 | 20.73 | 22.65 | 21.05 | 18.51 | Stage*Time: | 0.506 | 0.477 |  |
| 16.0 | Recent | 58.13 | 57.82 | 50.06 | 49.74 | 40.69 | 32.61 | Stage: | 4.819 | **0.028** | ***** |
|  | Long-term | 50.35 | 54.30 | 49.58 | 44.15 | 36.84 | 29.47 | Stage*Time: | 0.374 | 0.541 |  |
| 16.1ω9 | Recent | 9.17 | 11.62 | 22.95 | 23.77 | NA | NA | Stage: | 0.312 | 0.576 |  |
|  | Long-term | 3.36 | 12.59 | 21.00 | 20.29 | 21.69 | 20.06 | Stage*Time: | 28.091 | **<0.001** | ******* |
| 16.1ω7c | Recent | 44.66 | 55.95 | 44.73 | 42.44 | 34.06 | 28.97 | Stage: | 4.581 | **0.032** | ***** |
|  | Long-term | 29.72 | 41.92 | 38.49 | 33.07 | 28.85 | 25.51 | Stage*Time: | 9.814 | **0.002** | ****** |
| 16.1ω5 | Recent | 7.81 | 17.80 | 25.94 | 28.56 | 24.25 | 22.54 | Stage: | 0.859 | 0.354 |  |
|  | Long-term | 7.12 | 14.20 | 21.54 | 22.35 | 22.52 | 20.23 | Stage*Time: | 0.053 | 0.817 |  |
| 10Me16.0 | Recent | 0.85 | 5.83 | 8.54 | 13.71 | 14.33 | 15.71 | Stage: | 0.868 | 0.351 |  |
|  | Long-term | 0.43 | 4.32 | 8.90 | 10.98 | 12.22 | 12.85 | Stage*Time: | 1.359 | 0.244 |  |
| 17.0 | Recent | 27.69 | 30.33 | 28.62 | 33.99 | 28.92 | 25.21 | Stage: | 0.266 | 0.606 |  |
|  | Long-term | 23.68 | 28.38 | 31.88 | 32.08 | 26.93 | 21.22 | Stage*Time: | 0.522 | 0.470 |  |
| i17.0 | Recent | 5.58 | 15.16 | 22.59 | 29.45 | 27.74 | 24.46 | Stage: | 1.433 | 0.231 |  |
|  | Long-term | 5.13 | 12.18 | 19.42 | 22.91 | 22.24 | 19.76 | Stage*Time: | 0.687 | 0.407 |  |
| a17.0 | Recent | 21.89 | 24.00 | 20.58 | 28.91 | 23.36 | 21.47 | Stage: | 1.664 | 0.197 |  |
|  | Long-term | 13.66 | 18.12 | 18.79 | 20.27 | 17.49 | 17.23 | Stage*Time: | 0.167 | 0.683 |  |
| cy17.0 | Recent | 12.49 | 50.97 | 51.68 | 52.17 | 44.07 | 38.15 | Stage: | 3.824 | 0.051 | . |
|  | Long-term | 7.76 | 43.76 | 51.21 | 46.68 | 39.64 | 33.88 | Stage*Time: | NA | NA |  |
| 10Me17.0 | Recent | 2.57 | 11.59 | 11.81 | 17.42 | 17.83 | 18.70 | Stage: | 0.348 | 0.555 |  |
|  | Long-term | 1.35 | 7.01 | 11.36 | 14.01 | 14.55 | 13.49 | Stage*Time: | 4.625 | **0.032** | ***** |
| 18.0 | Recent | 30.05 | 32.13 | 24.44 | 34.29 | 23.89 | 21.69 | Stage: | 0.179 | 0.672 |  |
|  | Long-term | 33.93 | 33.03 | 30.12 | 29.50 | 22.99 | 18.97 | Stage*Time: | 0.815 | 0.367 |  |
| 10Me18.0 | Recent | 3.39 | 16.44 | 16.54 | 25.33 | 25.59 | 27.72 | Stage: | 0.734 | 0.392 |  |
|  | Long-term | -0.20 | 7.94 | 15.32 | 16.81 | 19.13 | 19.39 | Stage*Time: | 6.903 | **0.009** | ****** |
| 18.2ω6 | Recent | 97.79 | 101.03 | 89.17 | 87.63 | 80.60 | 70.38 | Stage: | 2.091 | 0.148 |  |
|  | Long-term | 87.73 | 96.83 | 91.82 | 81.31 | 76.20 | 66.78 | Stage*Time: | 0.093 | 0.763 |  |
| 18.1ω9 | Recent | 71.74 | 72.98 | 63.98 | 62.72 | 53.18 | 43.47 | Stage: | 5.415 | **0.020** | ***** |
|  | Long-term | 62.28 | 65.26 | 60.73 | 53.18 | 45.53 | 38.91 | Stage*Time: | 0.872 | 0.350 |  |
| 18.1ω7 | Recent | 35.48 | 49.33 | 45.07 | 44.02 | 38.45 | 35.23 | Stage: | 3.340 | 0.068 | . |
|  | Long-term | 31.40 | 40.34 | 41.30 | 37.63 | 33.65 | 30.86 | Stage*Time: | 4.648 | 0.199 |  |
| 20.4 | Recent | 17.42 | 34.43 | 38.51 | 51.18 | 49.11 | 52.95 | Stage: | 0.524 | 0.469 |  |
|  | Long-term | 17.80 | 37.34 | 43.03 | 46.29 | 50.10 | 47.64 | Stage*Time: | 0.163 | 0.686 |  |
| 20.5 | Recent | 21.89 | 36.11 | 45.78 | 53.10 | 47.77 | 53.90 | Stage: | 1.043 | 0.307 |  |
|  | Long-term | 22.00 | 40.23 | 43.99 | 53.23 | 56.27 | 54.58 | Stage*Time: | 0.478 | 0.489 |  |
| 20.0 | Recent | 45.22 | 32.28 | 24.45 | 31.34 | 24.88 | 15.40 | Stage: | 2.637 | 0.104 |  |
|  | Long-term | 39.43 | 34.18 | 23.61 | 24.94 | 16.40 | 9.53 | Stage*Time: | NA | NA |  |
